# Supplementary material for: Content-rich biological network constructed by mining PubMed abstracts
Source: BMC Bioinformatics. 2004 Oct 8;5:147. doi: 10.1186/1471-2105-5-147 (PMC528731; doi:10.1186/1471-2105-5-147)
Supplement: Additional File 2 — The original results of the above study (non-essential files are deleted to keep the file size under the limit set by BMC bioinformatics). [file 1471-2105-5-147-S2.bz2 › chilibotAdditionalFile2/dip50/108ID7559507E445/html/IGF1R_IRS1.html]

 


 **IGF1R** and **IRS1** 
  
Found 109 abstracts in PubMed, retrieved 50.  
 

 What does Google say? 
 PDF only 
| .edu only 

---

**Interactive relationship** (e.g. stimulation, inhibition, etc)

**Inhibitory relationship**- We also observed that silibinin reduced insulin receptor substrate 1  [ **IRS1** ]  tyrosine phosphorylation, indicating an inhibitory effect on the insulin like growth factor I receptor  [ **IGF1R** ]  mediated signaling pathway.  Ref: 11059749 Cancer Res, 2000
**Neutral relationship**- **Igf1r**  haploinsufficiency did not affect insulin action and compensatory beta cell growth in insulin resistant mice with combined Insr and  **Igf1r**  heterozygous null mutations, resulting in a considerably milder phenotype than combined haploinsufficiency for Insr and its main signaling substrates,  **Irs1**  and Irs2.  Ref: 12101187 J Biol Chem, 2002

**Non-interactive relationship** (e.g. studied together, co-existance, homology, etc.)

- We also measured  **IGF1R** , IRS 1  [ **IRS1** ] , and PTEN expression in 12 paired biopsies of primary prostate cancer and subsequent bone metastases.  Ref: 12019176 Cancer Res, 2002
- We investigated the expression pattern of insulin like growth factor I receptor  [ **IGF1R** ]  IGF IR, insulin receptor IR and insulin receptor substrate 1  [ **IRS1** ]  IRS 1  [ **IRS1** ] , a core downstream signaling protein, in 69 primary breast cancer specimens of different grades and in 21 control tissues by immunohistochemistry.  Ref: 11102895 Int J Cancer, 2000
- Estrogen can increase insulin like growth factor I receptor  [ **IGF1R** ]  IGF IR and insulin receptor substrate 1  [ **IRS1** ]  IRS 1  [ **IRS1** ]  expression, two key components of IGF I mediated signaling.  Ref: 11479214 Cancer Res, 2001
- We performed semiquantitative immunostaining for the  **IGF1R** , IRS 1  [ **IRS1** ] , and PTEN, and in situ hybridization for  **IGF1R** .  Ref: 12019176 Cancer Res, 2002
- Immunoprecipitation experiments showed that insulin like growth factor I receptor  [ **IGF1R** ]  and insulin receptor substrate 1  [ **IRS1** ]  were tyrosine phosphorylated to the same extent in both cells treated with colchicine and in those not exposed to the drug.  Ref: 10022515 J Cell Biochem, 1999
- In four cases,  **IGF1R**  and IRS 1  [ **IRS1** ]  levels were lower in the metastases than in the primary tumors.  Ref: 12019176 Cancer Res, 2002
- A receptor binding assay based on the surface plasmon resonance biosensor technique was developed to study the interaction between insulin like growth factor 1 receptor  [ **IGF1R** ]  and its intracellular substrate protein insulin receptor substrate 1  [ **IRS1** ] .  Ref: 11557037 FEBS Lett, 2001
- Down regulation of insulin like growth factor I receptor  [ **IGF1R** ]  and insulin receptor substrate 1  [ **IRS1** ]  expression in advanced human breast cancer.  Ref: 11102895 Int J Cancer, 2000
- Inhibition of insulin like growth factor 1 receptor  [ **IGF1R** ]  and IRS  [ **IRS1** ]  2 signaling by ethanol in SH SY5Y neuroblastoma cells.  Ref: 11208920 J Neurochem, 2001
- However, this pattern was relatively uncommon, and 8 of 12 cases expressed detectable  **IGF1R**  and IRS 1  [ **IRS1** ]  in both primary and metastatic biopsies.  Ref: 12019176 Cancer Res, 2002
- Recent studies in a human cell line and a mouse model suggest that metastatic prostate cancer cell detachment may be favored by impairing cadherin function via loss of expression of insulin receptor substrate 1  [ **IRS1** ] , the principal  **IGF1R**  docking molecule.  Ref: 12019176 Cancer Res, 2002
